# Participation in research improves overall patient management: insights from the Global Rheumatic Heart Disease registry (REMEDY)

**DOI:** 10.5830/CVJA-2017-054

**Published:** 2018

**Authors:** Prendergast EA, Perkins S, Joachim A, Zühlke LJ, Engel ME, Francis V, Mayosi B, Zühlke LJ, Cupido B, Mayosi B, Zühlke LJ, Al Kebsi M, Bode-Thomas F, Damasceno A, Abul Fadl A, El Sayed A, Ibrahim A, Gitura B, Kennedy N, Mucumbitsi J, Adeoye AM, Musuku J, Okello E, Olunuga T, Sheta S, Mayosi BM

**Affiliations:** Department of Paediatrics and Child Health, Red Cross War Memorial Children’s Hospital, Faculty of Health Sciences, University of Cape Town, Cape Town, South Africa; Department of Paediatrics and Child Health, Red Cross War Memorial Children’s Hospital, Faculty of Health Sciences, University of Cape Town, Cape Town, South Africa; Department of Paediatrics and Child Health, Red Cross War Memorial Children’s Hospital, Faculty of Health Sciences, University of Cape Town, Cape Town, South Africa; Department of Paediatrics and Child Health, Red Cross War Memorial Children’s Hospital, Faculty of Health Sciences, University of Cape Town, Cape Town, South Africa; Department of Medicine, Groote Schuur Hospital, Faculty of Health Sciences, University of Cape Town, Cape Town, South Africa; Department of Medicine, Groote Schuur Hospital, Faculty of Health Sciences, University of Cape Town, Cape Town, South Africa; Department of Medicine, Groote Schuur Hospital, Faculty of Health Sciences, University of Cape Town, Cape Town, South Africa; Department of Medicine, Groote Schuur Hospital, Faculty of Health Sciences, University of Cape Town, Cape Town, South Africa; Division of Cardiology, Groote Schuur Hospital, Faculty of Health Sciences, University of Cape Town, Cape Town, South Africa; Division of Cardiology, Groote Schuur Hospital, Faculty of Health Sciences, University of Cape Town, Cape Town, South Africa; Division of Cardiology, Groote Schuur Hospital, Faculty of Health Sciences, University of Cape Town, Cape Town, South Africa; Faculty of Medicine and Surgery, University of Sana’a, Al–Thawrah, Cardiac Centre, Sana’a, Yemen; Departments of Paediatrics, University of Jos and Jos University Teaching Hospital, Jos, Nigeria; Department of Medicine, Eduardo Mondlane University, Maputo, Mozambique; Faculty of Medicine, Benha University, Cairo, Egypt; Cardiothoracic Surgery Department, Al Shaab Teaching Hospital and Faculty of Medicine, Alzaiem Alazhari University, Khartoum, Sudan; Cardiothoracic Surgery Department, Al Shaab Teaching Hospital and Faculty of Medicine, Alzaiem Alazhari University, Khartoum, Sudan; Cardiology Unit, Department of Medicine, Kenyatta National Teaching and Referral Hospital, Nairobi, Kenya; Department of Paediatrics and Child Health, College of Medicine, University of Malawi, Blantyre, Malawi; Centre for Medical Education, Queen’s University, Belfast; Royal Belfast Hospital for Sick Children, Belfast, Ireland; Paediatric Cardiology Unit, Department of Paediatrics, King Faisal Hospital, Kigali, Rwanda; Division of Cardiology, Department of Medicine, University College Hospital, Ibadan, Nigeria; University Teaching Hospital, Department of Paediatrics and Child Health, University of Zambia, Lusaka, Zambia; Uganda Heart Institute, Kampala, Uganda; Department of Medicine, Federal Medical Centre, Abeokuta, Nigeria; Department of Paediatrics, Division of Paediatric Cardiology, Faculty of Medicine, Cairo University Children’s Hospital, Cairo, Egypt; Dean of Faculty of Health Sciences, University of Cape Town, South Africa

**Keywords:** rheumatic heart disease, REMEDY study, clinical research, low- and middle-income countries, implementation

## Abstract

**Background:**

Rheumatic heart disease (RHD) is a major public health problem in low– and middle–income countries (LMICs), with a paucity of high–quality trial data to improve patient outcomes. Investigators felt that involvement in a recent large, observational RHD study impacted positively on their practice, but this was poorly defined.

**Aim:**

The purpose of this study was to document the experience of investigators and research team members from LMICs who participated in a prospective, multi–centre study, the global Rheumatic Heart Disease Registry (REMEDY), conducted in 25 centres in 14 countries from 2010 to 2012.

**Methods:**

We conducted an online survey of site personnel to identify and quantify their experiences. Telephone interviews were conducted with a subset of respondents to gather additional qualitative data. We asked about their experiences, positive and negative, and about any changes in RHD management practices resulting from their participation in REMEDY as a registry site.

**Results:**

The majority of respondents in both the survey and telephone interviews indicated that participation as a registry site improved their management of RHD patients. Administrative changes included increased attention to follow–up appointments and details in patient records. Clinical changes included increased use of penicillin prophylaxis, and more frequent INR monitoring and contraceptive counselling.

**Conclusions:**

Our study demonstrates that participation in clinical research on RHD can have a positive impact on patient management. Furthermore, REMEDY has led to increased patient awareness and improved healthcare workers’ knowledge and efficiency in caring for RHD patients.

Rheumatic heart disease (RHD) is the principal cause of valvular heart disease–related mortality and morbidity in lowand middle–income countries (LMICs). It predominantly affects children and young adults and is potentially responsible for approximately 233 000 deaths per year worldwide.^1^ However, contemporary data documenting the presentation, clinical course, complications, and ‘real–world’ treatment of RHD are relatively scarce.

The Global Rheumatic Heart Disease Registry (REMEDY) was a prospective registry of 3 343 patients with RHD from 25 sites in 14 LMICs that was conducted from January 2010 to November 2012.^2^ It documented both clinical and echocardiographic characteristics of the patients, and outcomes and current treatment practices, with particular reference to adherence to secondary prophylaxis with penicillin and oral anticoagulation regimens.^3^,^4^

The outcomes of REMEDY have drawn attention to a number of concerns. First, although patients were young, two–year case fatality rate was high.^4^ Second, post–primary school education level is associated with lower risk of death, and third, patients from low– and lower–middle–income countries have higher ageand gender–adjusted mortality rates than patients from uppermiddle– income countries. Fourth, valve surgery is more frequently undertaken in upper–middle–income countries than in low– and lower–middle–income countries. These findings have motivated further research and changes in clinical practice relating to RHD at many of the original REMEDY investigation sites.^5^–^8^

It is well known that clinical outcomes of patients who participate in clinical research are superior to those in realworld practice.^9^ In randomised trials, this effect may relate to selective enrolment but the explanations in registry studies are poorly defined. Therefore, our study aimed to identify the major challenges and opportunities encountered by investigators and members of the research teams during the study and to provide a useful reference for researchers working on future similar projects in LMICs.

## Methods

We created an online survey comprising four sections, with a total of 45 questions (Table 1). The online survey addressed questions concerning patient follow up, administration and clinical management, staff training, and on–site resources. The majority of questions (34/45) followed a multiple–choice format to quantify the challenges and opportunities encountered by investigators during the study.

**Table 1:** Challenges and opportunities: the REMEDY study (online survey)

**Section 1: Personal details**
1. Name
2. Role
3. Site/centre number
4. Name of hospital/facility
5. Country
6. Would you be interested in participating in the telephone interview?
7. If yes, how would you prefer to be contacted?
8. Please specify your contact details and best time/day for calling
**Section 2: About your site**
9. How would you describe your site?
10. Before REMEDY, had your site ever conducted a local/single-site research study before?
11. Before REMEDY, had your site ever conducted a multi-centre research study before?
12. How many members of staff on your site participated in REMEDY?
13. Was a GCP course offered on-site?
14. If yes, how many members of staff completed a GCP course?
15. If no, did any of your staff complete GCP training as part of REMEDY?
16. Did you attend a REMEDY investigator meeting?
17. If yes, how far do you agree with the following statement? ‘The investigator meeting was productive and supportive, providing an opportunity for learning and clarification. Adequate time was provided to give and receive feedback. I felt confident to continue with the conduct of the study after the meeting.’
18. Did you have a site initiation visit from a representative from the UCT project coordination office?
19. If yes, how useful did you find it? ‘I felt I was given complete information and adequate time to learn and ask questions. I was confident to conduct the study at the end of the visit.’
20. Did you have an on-site monitoring visit from a representative from the UCT project coordination office?
21. If yes, how useful did you find it? ‘The visit was productive and supportive, providing an opportunity for learning and clarification. Adequate time was provided to give and receive feedback. I felt confident to continue with the conduct of the study after the visit.’
22. What would you change about the training you received?
**Section 3: Organisation and accessibility**
23. Was INR available on-site?
24. If yes, on-site INR results were generally available
25. Results were made available by
26. If not available on-site, where was testing performed?
27. If not available on-site, how long did it take to receive results?
28. What supplies/equipment did you purchase specifically for conducting the REMEDY study?
29. What type of echo equipment did you use?
30. Did you have easy access to an ECG machine?
31. Did you have access to ECG paper?
32. CRFs were sent to the UCT project coordination office
33. During the study, my access to REMEDY email and internet was
34. During the study, medical records at my site were
35. Did you change the way you manage your RHD patients as a result of participating in the REMEDY study?
36. Please check any administrative changes due to the REMEDY study
37. Please check any clinical changes due to the REMEDY study
**Section 4: Patients**
38. Where were baseline ECGs conducted?
39. Where were baseline echos conducted?
40. Did your site experience stock-out problems for penicillin during the study?
41. Did your site experience stock-out problems for anticoagulants during the study?
42. Did your site experience stock-out problems for other cardiac drugs during the study?
43. What were the most difficult challenges you faced upon following up patients?
44. Did you experience any other challenges not mentioned above upon following up patients?
45. How did you resolve them?

**Fig. 1:**
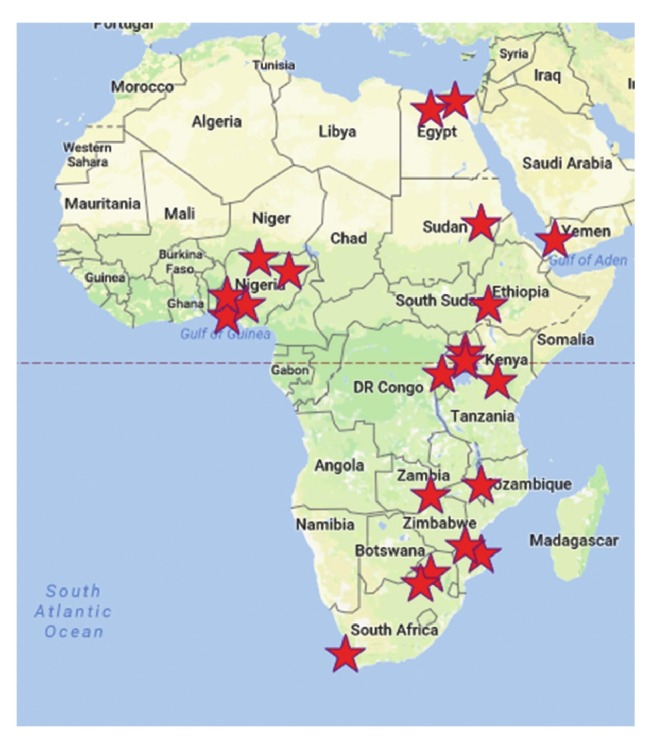
Participants from 22 sites completed the survey.

**Fig. 2:**
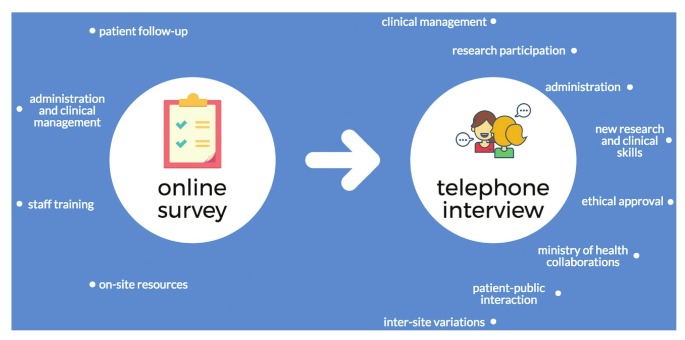
Information-gathering methods and their components.

Thirty participants from 22 sites completed the survey (Fig. 1). Most survey respondents (19/30) also participated in a followup telephonic interview (conducted by Skype, telephone or WhatsApp call) comprising 10 qualitative questions concerning their experiences during the study (Table 2). The telephonic questionnaire focused on clinical management, research participation, administration, research and clinical skills, ministry of health collaborations, patient–public interactions, inter–site variations and ethics approvals (Fig. 2).

**Table 2:** Challenges and opportunities: the REMEDY study

**Telephone interview questions**
1. For many of the REMEDY sites, it was their first time participating in a multi-centre research project. Was this the case for your site?
2. Some participants have said that REMEDY had an impact on the clinical, research, academic and administrative aspects of their sites. For example, some reported that it changed the way in which they ran clinics. What impact did REMEDY have on your site?
3. Were you able to obtain additional resources by the fact that you were in the REMEDY study?
4. Did REMEDY have any impact on your relationship with the Ministry of Health?
5. Some members of staff have said that they acquired some skills as a result of the REMEDY study. Was this the case at your site?
6. Have you/they used these skills in other contexts?
7. Would you have liked an opportunity to learn anything else during the study?
8. What was your experience with ethics and institutional approval processes?
9. What, if any, impact has REMEDY had on your RHD patients?
10. Is there anything else that you would like to tell me that REMEDY did or did not do for your site?

## Results

**Online survey**

**Fig. 3:**
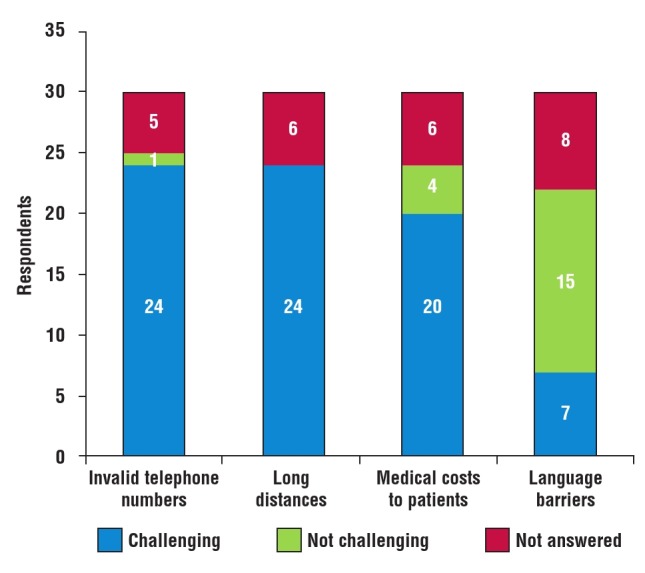
Common barriers to follow up.

Patient follow up: all respondents (30/30) experienced significant difficulties with the follow up of their RHD patients. Participants identified invalid telephone numbers (24/30), long distances (24/30), medical costs to patients (20/30) and language barriers (7/30) as common problems (Fig. 3). Other barriers included late ethics committee approvals, lack of study funding, lack of support from other on–site staff and difficulty in tracing patients’ addresses.

Strategies to reduce losses to follow up included initiation of home visits to patients who had missed appointments, collection of several telephone numbers from patients and relatives, initiation of telephone reminders before clinics, sketching patient residences on a map in the absence of a formal address system and educating patients about the importance of regular follow up.

**Fig. 4:**
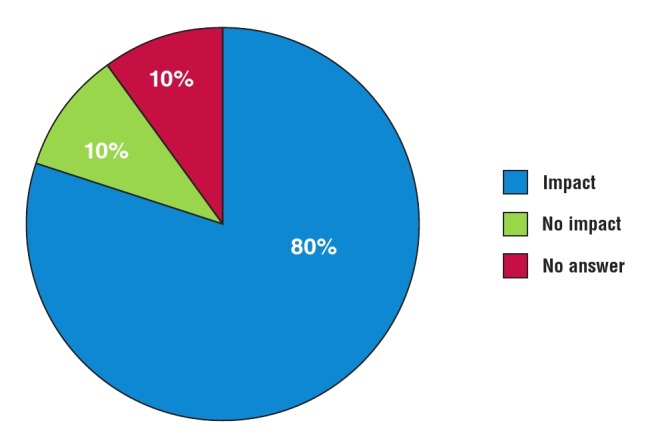
Online survey: impact of REMEDY on patient management.

Administration and clinical management: the majority of responses (24/30) were positive when asked whether participation in REMEDY changed their management of RHD patients (Fig. 4). Administrative changes included increased frequency of follow–up appointments (14/24), increased information noted in patient records (13/24), and changes to clinic times and booking systems (6/24). Clinical changes included more rigorous prescribing practices for penicillin prophylaxis (15/24) and warfarin (6/24), more frequent international normalised ratio (INR) monitoring (11/24), and increased efforts to provide contraceptive counselling to post–menarchal females (9/24).

Staff training: in total, 8/30 respondents’ sites offered a good clinical practice (GCP) course on–site that was completed by the majority of staff at 5/8 sites. On–site GCP training was unavailable to 18/30 respondents. Nevertheless, 10/18 respondents stated that staff completed GCP courses via other mechanisms, such as online courses.

Twenty–four/30 respondents attended a REMEDY investigator meeting; 21/24 agreed that the meeting was productive and supportive, that adequate time was provided to give and receive feedback and that they felt confident to continue with the study after the meeting. Ten/30 respondents received a site initiation visit from a representative of the project coordination office (PCO); 10/10 agreed that they were given adequate information and time to learn during the visit and that they felt confident to conduct the study afterwards. Thirteen/30 received an on–site monitoring visit from a representative of the PCO. Of these, 12/13 agreed that the visit was productive and supportive, provided opportunity for learning, clarification and feedback, and increased their confidence to continue with the study.

When asked whether they would change anything about the training they received, most (19/30) respondents did not answer, 3/30 stated that they would not change anything and 8/30 made suggestions for future related studies that included clarification about specific medical terminology, drug categories and diagnostic tests, increased numbers of investigator meetings and monitoring visits, mandatory GCP courses and increased online communication.

**Fig. 5:**
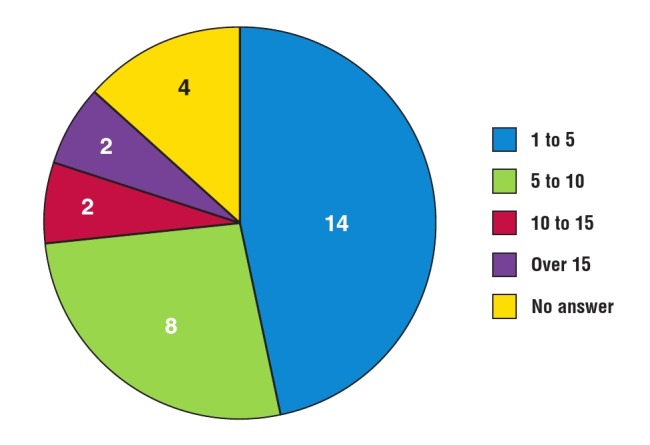
Online survey: numbers of on-site REMEDY staff.

On–site resources: most (26/30) respondents’ sites had participated in single–site research before REMEDY. Most (20/26) had also participated in multi–centre research. As a result, different sites had different capacities to conduct research over two years. For example, numbers of staff greatly varied across REMEDY sites. Fourteen/30 respondents’ teams comprised one to five individuals, 8/30 comprised five to 10 individuals, 2/30 comprised 10 to 15 individuals, and 2/30 had over 15 members of staff dedicated to the project (Fig. 5).

INR monitoring was available on 23/30 respondents’ sites. On–site INR results were available at point of care (5/23), on the same day as patient visits (9/23), after visits (6/23) or at times not specified on the survey, such as the day before visits (3/23). Results were made available by telephone (4/23), hardcopy printouts (16/23) or electronic devices (1/23). INR was not available on–site for 7/30 respondents and instead was performed at nearby hospitals, private laboratories or non–governmental organisation–run clinics. Off–site results were received on the same day as patient visits (1/7), between one and seven days after visits (5/7) or over seven days after visits (1/7).

The majority of sites had access to adequate resources for conducting electrocardiograms (ECGs) and echocardiograms (echos). Baseline echos were conducted on–site at 27/30 respondents’ sites and baseline ECGs were conducted on–site at 26/30 sites. While 26/30 respondents always or usually had access to ECG machines, 2/30 sometimes or seldom had access. Twentyfive/ 30 respondents always or usually had access to ECG paper while 2/30 sometimes or seldom had access.

**Fig. 6:**
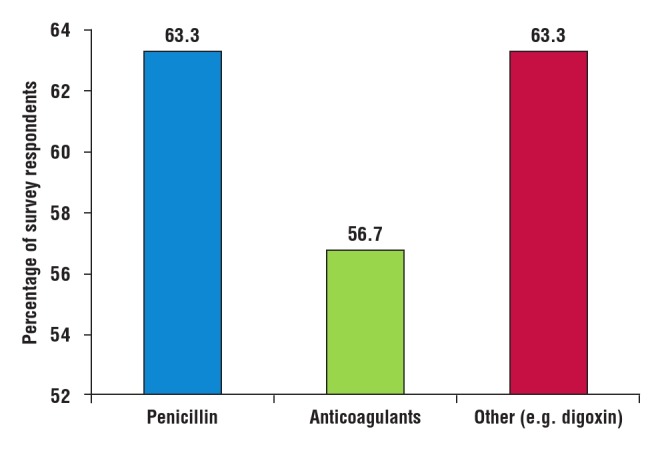
Online survey: drug stock-out problems.

The majority of respondents experienced drug stock–out problems for their RHD patients, including penicillin (19/30), anticoagulants (17/30) and other cardiac drugs such as digoxin, ACE inhibitors, spironolactone and captopril (19/30) (Fig. 6).

On–site internet access varied across sites. For example, REMEDY e–mail was either provided by respondents’ work facilities (15/30) or by personal devices and funds (13/30) throughout the study.

**Fig. 7:**
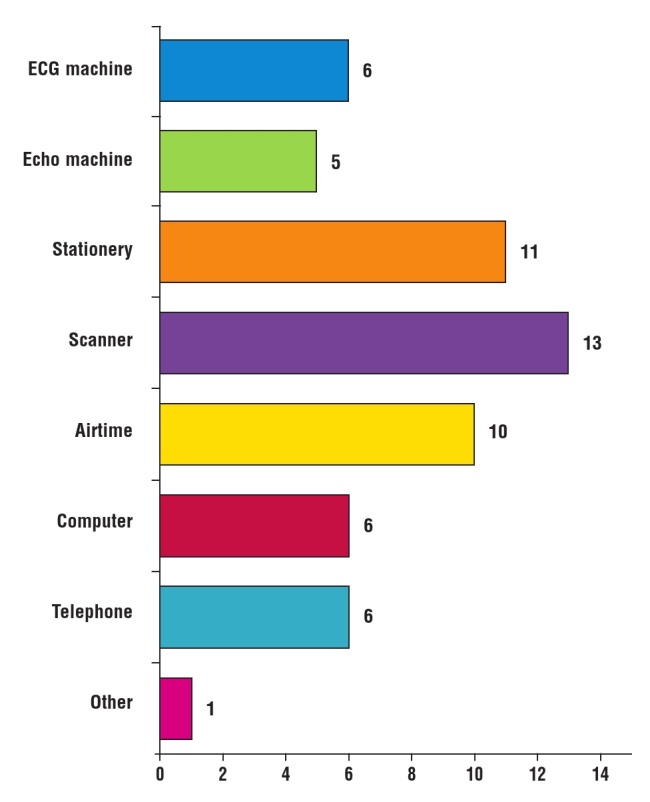
Online survey: items purchased during REMEDY.

Several sites purchased supplies for conducting the REMEDY study. Items bought included telephones (6/30), computers (6/30), airtime (10/30), scanners, copiers and fax machines (13/30), patient binders, files and stationery (11/30), echo machines (5/30), ECG machines (6/30) and other supplies not mentioned in the survey (1/30) such as furniture. Seven/30 respondents did not purchase anything for conducting REMEDY (Fig. 7).

## Telephone interview

**Fig. 8:**
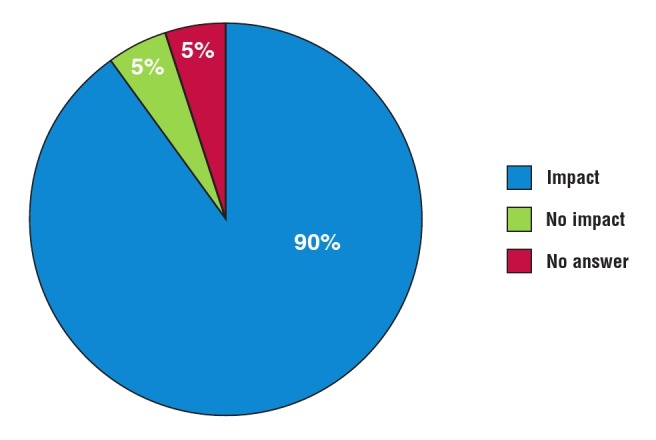
Telephone interview: impact of REMEDY on patient management.

Clinical management: almost all responses (17/19) were positive when asked whether participation in REMEDY changed their management of RHD patients (Fig. 8). Changes included more rigorous use of penicillin prophylaxis and anticoagulation, increased efforts to reduce loss to follow up, establishment of independent RHD clinics, more regular INR management, higher–quality standards for echocardiography, improved knowledge concerning early symptoms of RHD, and increased efforts to provide family planning counselling to post–menarchal females. For example, one participant remarked, ‘Before REMEDY, we were not very keen on important interventions like family planning and mandatory injections. REMEDY led us to be more vigilant, to encourage family planning and to make sure our RHD patients are getting regular medications. It has improved the care for these patients’.

Research participation: for 15/19 respondents, REMEDY encouraged further participation in rheumatic and congenital heart disease projects and collaboration with researchers in these fields. At least eight sites have continued working with REMEDY investigators on subsequent studies (INVICTUS, RHDGen and Afrostrep) while independent sub–projects have focused on pre–school screening for RHD, atrial fibrillation, primary prevention measures for RHD, and co–morbid associations with hepatitis B.^10^

Administration: results varied when participants were asked whether participation in REMEDY changed administrative structures at their sites. Some (5/19) stated that it changed systems for the filing of patient records and recording the echocardiographic findings, 9/19 stated that it had no impact and 5/19 did not comment on its effect.

New research and clinical skills: approximately two–thirds of respondents (14/19) acquired research skills as a result of REMEDY, such as protocol preparation, data management and creation of case report forms, while 8/19 acquired new clinical skills, such as improved interpretation of echocardiograms. The vast majority (16/19) used these skills subsequently in other contexts. Moreover, 14/19 remarked that they would have valued the opportunity to learn further skills during the study. One researcher suggested that their site would have benefitted from an introductory research course to familiarise the research team with the chronology of the study, required steps and study timelines. Other suggestions included increased site–initiation training, on–site visits to ensure quality control, statistical analysis courses, increased guidance on how to perform echocardiography, and more training in anticoagulant management.

Ministry of Health collaborations: for 11/19 researchers, REMEDY had an impact on their site’s relationship with the Ministry of Health. One investigator shared their site’s data with the national Ministry of Health in order to procure assistance in drawing up guidelines for the detection and prevention of RHD. At a different site, the findings of REMEDY led to the creation of a national RHD registry and investment in echocardiography machines by the local Ministry of Health. Another site used REMEDY results to collaborate with the Ministry of Health in securing approval for RHD screening in schools.

**Fig. 9:**
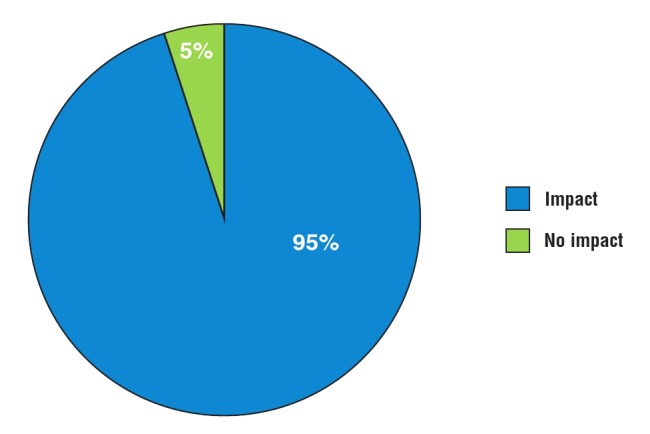
Telephone interview: impact of REMEDY on patient behaviour.

Patient–public interaction: almost all participants (18/19) responded positively when asked whether participation in REMEDY had an impact on interaction with their RHD patients (Fig. 9). For example, investigators at the Groote Schuur and Red Cross War Memorial Children’s Hospitals (Cape Town) presented the outcomes of REMEDY to their RHD patients in 2014. The hospital now hosts an annual event for its RHD patients, which aims to empower patients by improving their understanding of the disease and compliance with treatment.

Additionally, a patient community advisory board has been established as a community liaison between patients and clinical researchers. Similarly, the Uganda Heart Institute established a patient support group in which long–term RHD patients support clinicians in counselling newly diagnosed patients. Such groups build patient confidence and empower them to manage their disease.

Inter–site variations: Although the telephone findings were largely consistent across study sites, wide variation in previous exposure to multi–centre research, patient volume, and access to healthcare resources provided a unique set of challenges and opportunities at each centre. For example, 11/16 respondents who had previously participated in multi–centre research (and 9/11 who had already conducted specific cardiology research) employed pre–existing administrative, human and material resources during REMEDY.

Furthermore, on–site support for REMEDY varied greatly from one site to the next, with team sizes ranging from 20 members of staff to one principal investigator acting alone. Those with limited support found the study taxing on their time and resources and expressed that REMEDY had limited impact on their site as a result. Several methods were employed by investigators to resolve this challenge. For example, 8/19 sought additional resources from non–governmental organisations, other hospitals and pharmaceutical companies. One site established a research committee with the intent of employing full–time administrative staff to relieve pressure experienced by clinicians in the course of research activities.

Ethical approval: given that REMEDY is a prospective, non–interventional registry, 13/19 sites experienced little difficulty in obtaining ethical clearance. Those that experienced difficulties were unable to recruit large numbers of patients for the study, demonstrating the importance of early application for ethical and institutional approval.

## Principal investigator responses

The principal investigators of REMEDY were asked an additional question about unexpected challenges during the course of the study. A common problem was the variation in availability of patient identifiers (such as date of birth, thumbprints and formal addresses) across sites. Similarly, clinical records for each patient’s history of stroke, HIV and contraceptive use differed across sites, leading to underestimation of their incidence.

Political situations also impacted on the study’s progress. Halfway through the study, South Sudan became an independent nation, resulting in a 30% loss of Sudanese patients from the registry. Furthermore, the civil war in Yemen and the Arab Spring unrest in Cairo significantly hindered follow up of REMEDY patients in these countries.

Both principal investigators therefore stressed the importance of start–up and progress meetings in the course of a multicentre research project. Although challenging to implement, monitoring and evaluation strategies allow investigators to identify and address challenges encountered by individual sites. For example, a monitoring visit to Zambia allowed the Project Coordination Office to update REMEDY patient files on site, while visits to other sites resulted in grants for fax machines, cellphone data and laptop computers to enable the transmission of REMEDY data to Cape Town.

## Discussion

The impact of REMEDY on everyday practice in a variety of geographical and clinical settings demonstrates that RHD research has a positive effect on patient management. Clinical practices established during REMEDY, such as closer follow up, increased provision of family planning counselling and the promulgation of independent RHD clinics have continued since original publication of the study. For example, one participant remarked, ‘Before REMEDY we didn’t realise what proportion of patients stopped coming for follow up. Some of them were not regular in attending their clinic and nobody really noticed, but because we had to keep track of them during REMEDY, it improved their engagement with the healthcare system and their follow–up attendance’.

Involvement in REMEDY also resulted in the acquisition of new research skills by the study team members and improved sites’ ability to conduct RHD research, as demonstrated by the increase in RHD projects post–REMEDY. Furthermore, publication of the results of REMEDY^3^,^4^,^11^ increased public awareness of RHD and advocacy for its prevention among higher medical and political authorities, such as local and national ministries of health.

Involvement in a project with such widespread impact boosted morale among staff at sites with high volumes of RHD patients. For example, one researcher commented, ‘When you’re faced with tides and tides of patients and you’re on your own, it can feel quite disheartening. REMEDY encouraged participants, it gave job satisfaction and it improved motivation in general’.

Several factors impeded the study’s progress. Unavoidable situations such as informal address systems and political unrest in several countries where REMEDY sites were based resulted in loss to follow up of REMEDY patients. Furthermore, the majority of survey respondents (22/30) had small on–site teams (0–10 individuals) to assist them in conducting the REMEDY study. Those with limited on–site support remarked in the telephone interview that they found the study taxing on their time and resources and that, as a result, they were unable to perform critical tasks such as timely application for ethical approval. On–site support for GCP training courses was also lacking for 18/30 survey respondents. Given the positive feedback and advocacy from both survey and telephone interview respondents for more GCP training, site initiation and monitoring visits, future related projects should dedicate time and funding to on–site visits and training in order to educate investigators about the project and to assess each site’s resources individually.

Our study has several implications for future research and clinical practice. First, the rise in rheumatic and congenital heart disease projects and ensuing collaboration among cardiologists as a result of REMEDY has greatly increased global awareness of RHD. This growing research network is a major advocacy tool for the disease and demonstrates the importance of continuing efforts to conduct and facilitate RHD research.

Second, our results indicate that observational registries such as REMEDY have significant value. Not only did the publication of the findings of REMEDY increase public awareness of RHD but it also directly improved clinicians’ and patients’ understanding of the disease. In resource–limited countries, the initiation of local and national registries is the cornerstone of the RHD prevention and control programmes recommended by the World Health Organisation and the World Heart Federation.

Finally, the majority of survey and telephone interview respondents used their experiences during REMEDY to propose suggestions for future related studies. Their ideas provide a valuable resource to researchers working on similar projects and demonstrate the importance of involving clinicians who are active in the field (and not just those in principal academic centres) in programmes of clinical research.

Our study highlights these important implications but is of course limited by the subjective impression from investigators and research staff, rather than stringent monitoring and evaluation processes running alongside the original study. We suggest therefore that these are incorporated into future studies in LMICs to demonstrate the additional benefits (or disadvantages) of research to communities, research personnel and patients.

## Conclusions

Researchers in the field should draw confidence from our findings that RHD research improves overall patient management and advocacy for the disease. The important lessons learnt were strategies employed by the REMEDY investigators to reduce loss to follow up, the benefits of early application for ethics approval, and the importance of on–site initiation and monitoring during multi–centre projects.
